# Real-world care patterns and specialist encounters of patients with systemic autoimmune rheumatic disease-related interstitial lung disease in the United States: a retrospective administrative claims database analysis

**DOI:** 10.1093/rheumatology/keaf200

**Published:** 2025-04-23

**Authors:** Joseph Yang, Michael A Head, Kevin D Schott, Hiangkiat Tan, Rachel Djaraher, Christopher L Crowe, Mark Napier, Amy L Olson, Elana J Bernstein

**Affiliations:** Boehringer Ingelheim Pharmaceuticals, Inc, Ridgefield, CT, USA; HEOR, Carelon Research, Wilmington, DE, USA; HEOR, Carelon Research, Wilmington, DE, USA; HEOR, Carelon Research, Wilmington, DE, USA; HEOR, Carelon Research, Wilmington, DE, USA; HEOR, Carelon Research, Wilmington, DE, USA; Health and Wellness, Elevance Health, Inc, Latham, NY, USA; Boehringer Ingelheim Pharmaceuticals, Inc, Ridgefield, CT, USA; Division of Rheumatology, Department of Medicine, Vagelos College of Physicians and Surgeons, Columbia University Irving Medical Center, New York, NY, USA

**Keywords:** systemic autoimmune rheumatic diseases, interstitial lung disease, diagnosis, care patterns

## Abstract

**Objectives:**

We aimed to describe the demographics, clinical characteristics and care patterns of patients with systemic autoimmune rheumatic diseases (SARD) prior to their interstitial lung disease (ILD) diagnosis.

**Methods:**

We conducted a retrospective cohort study using claims data from Healthcare Integrated Research Database (2006–2023). Adults diagnosed with SARD-ILD were identified, with the earliest ILD diagnosis date designated as the index date. A minimum of 36 months of continuous enrolment before the index date was required. All measures were analysed descriptively. For a subset of patients with respiratory symptoms before ILD diagnosis, the association between type of specialist encounter and time from symptom onset to SARD-ILD diagnosis was assessed using a Cox proportional hazards model.

**Results:**

The study included 2526 patients with SARD-ILD. Mean age was 62.6 years and 75.4% were female. Before ILD diagnosis, 61.8% of patients had at least one all-cause hospitalization. Diagnostic tests including chest CT, high-resolution CT, and pulmonary function tests (PFT) were used in 80.1%, 59.0% and 60.3% of patients, respectively. Among the subgroup, patients who saw a pulmonologist within 90 days of initial respiratory symptom onset were 18% more likely to be diagnosed with ILD compared with those who did not (hazard ratio: 1.18, 95% CI: 1.03, 1.35; *P* = 0.017).

**Conclusion:**

The study highlights the complex diagnostic journey of patients with SARD-ILD. Findings suggest a multidisciplinary approach involving pulmonologists and rheumatologists could enable timely ILD diagnosis and should be considered for more effective diagnosis and management of SARD-ILD.

Rheumatology key messages

- The complications of early recognition of ILD may result in delays to diagnosis and treatment initiation.
- Common ILD diagnostic testing might be underutilized during the pre-index period, leading to delays in diagnosis.
- Multidisciplinary teams including pulmonologists and rheumatologists may lead to earlier SARD-ILD diagnosis in symptomatic patients.

## Introduction

Interstitial lung disease (ILD) is a broad term used to describe a group of disorders characterized by chronic inflammation and fibrosis of the lung interstitium [1]. Systemic autoimmune rheumatic diseases (SARDs) are a group of disorders that affect multiple organ systems, including the lungs. The development of ILD in the context of a SARD is known as SARD-ILD. Different SARDs have varying degrees of association with ILD. For example, systemic sclerosis (SSc), rheumatoid arthritis (RA), and the idiopathic inflammatory myopathies such as dermatomyositis (DM) and polymyositis (PM) are strongly associated with ILD, while systemic lupus erythematosus (SLE) is less commonly associated with ILD [2–5].

The pathogenesis of SARD-ILD is complex [6]. The clinical presentation of SARD-ILD is heterogeneous and non-specific, and patients with SARD-ILD commonly experience symptoms such as exertional dyspnoea, dry cough, fatigue and chest discomfort, which may overlap with other chronic comorbidities. Thus, patients with SARD-ILD often have a considerable diagnostic delay, and patients may be exposed to unnecessary and ineffective treatments in the interim [7].

Early and accurate diagnosis of SARD-ILD is vital for preserving lung function and improving patient outcomes. Given the complexity of SARD-ILD, early involvement of a team of specialists on a patient’s disease journey is crucial [8]. A multidisciplinary approach to chronic disease management has been shown to significantly benefit patients. The benefits include improved patient outcomes, streamlined workflow, and enhanced patient satisfaction [9, 10]. Such an approach could enable the team to diagnose and manage an individualized treatment plan collaboratively, thereby optimizing patient care and outcomes. Moreover, utilization of necessary diagnostic tests, including high-resolution CT (HRCT) scans of the chest and pulmonary function tests (PFT), in conjunction with a multidisciplinary approach, can enhance the overall diagnostic process and management of SARD-ILD [11–13]. Therefore, effective screening, diagnosis and management strategies can help prompt initiation of appropriate therapies and avoid unnecessary healthcare resource utilization (HCRU) [14].

Despite the importance of early diagnosis of SARD-ILD, few studies have evaluated the patient journey to ILD diagnosis among patients with SARDs. This study aims to fill this knowledge gap by better understanding the demographic and clinical characteristics of these patients and describing current care patterns prior to SARD-ILD diagnosis. Among a subset of patients with early ILD-related respiratory symptoms (dyspnoea and/or cough) between the initial SARD diagnosis and subsequent ILD diagnosis, we investigated the association between the timing of specialist encounters and the time from the onset of early respiratory symptoms to SARD-ILD diagnosis.

## Methods

### Study design and data

We performed a retrospective administrative claims database study of a cohort of adult patients with SARD-ILD and assessed their care patterns prior to ILD diagnosis. We used the Healthcare Integrated Research Database (HIRD^®^), a geographically diverse, extensive repository of longitudinal medical and pharmaceutical claims data, for this study. The data are sourced from health plans affiliated with Elevance Health, which represents over 65 million lives from both commercially insured and Medicare Advantage plans across all 50 states in the United States. Previous evaluations of the HIRD have shown that it is generally representative of the US Census population in terms of age and sex, although it slightly under-represents patients aged 65 years and older [15]. This study involved no direct contact with patients and used only de-identified data, and was therefore exempt from Institutional Review Board review.

**Figure 1.**
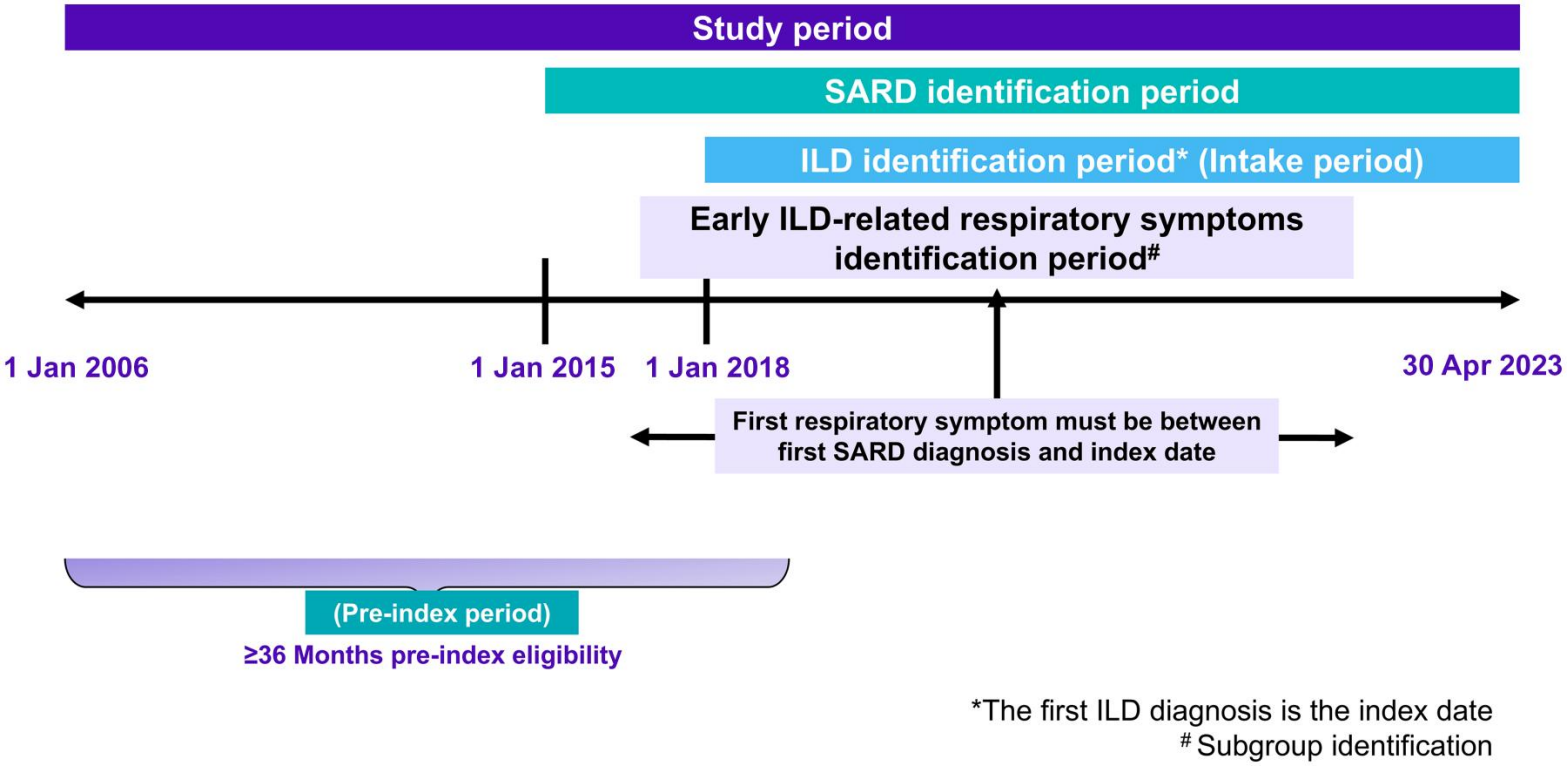
Study design and time periods. Detailed schematic illustration of the study design and time periods used to assess care patterns of patients with SARD-ILD prior to their ILD diagnoses. SARD: systemic autoimmune rheumatic disease; ILD: interstitial lung disease

The study period extended from 1 January 2006 to 30 April 2023. The study cohort consists of adults aged 18 years and older, newly diagnosed with SARD-ILD. First, we identified adult patients diagnosed with a SARD from 1 January 2015 to 30 April 2023 (SARD identification period). A SARD diagnosis was defined as the presence of two or more claims with International Classification of Diseases, Ninth Revision, Clinical Modification (ICD-9-CM) or International Classification of Diseases, Tenth Revision, Clinical Modification (ICD-10-CM) diagnosis codes within 1 year indicating the following conditions during the SARD identification period: ANCA-associated vasculitis, DM/PM, mixed connective tissue disease (MCTD), RA, Sjögren’s disease, SSc or SLE. Next, patients with two or more claims with an ICD-9/10-CM diagnosis for ILD within 1 year between 1 January 2018 and 30 April 2023 (ILD identification period) were included. The earliest observed date of claim with ILD diagnosis was designated as the index date. Patients were excluded from the study if they had an ILD diagnosis or antifibrotics utilization before the index date, as far back as 2006 as enrolment allows, or if the index date occurred before the initial SARD diagnosis date because the goal of this study was to understand the care patterns after SARD diagnosis leading to ILD diagnosis. Patients were required to have a minimum of 36 continuous months of medical and pharmaceutical enrolment prior to the index date (pre-index period), inclusive of the index date (Fig. 1). A minimum of 36 months of continuous enrolment was selected to provide a sufficient pre-index period to measure care patterns leading to ILD diagnosis.

The subgroup of patients with early respiratory symptoms related to ILD, defined as having one or more ICD-9/10-CM diagnosis code indicating cough and/or dyspnoea following SARD diagnosis and occurring at least 90 days prior to and exclusive of the index date, was identified from the study cohort.

### Demographics, clinical characteristics and outcomes

In this study, clinical characteristics and care patterns, including HCRU and specialist encounters, were measured and reported during the pre-index period. Patient demographics and social determinants of health (SDOH) variables were measured on the index date. Demographic information included age, sex, race/ethnicity and insurance payor types. SDOH variables were measured at the census block group level, including the quartiles of the socioeconomic status (SES) index score, income level, education level and unemployment rates.

Clinical characteristics were measured over the minimum 36-month pre-index period, including the underlying SARD types, respiratory and non-respiratory comorbidities, Quan–Charlson comorbidity index score (QCI) and ILD-related respiratory symptoms.

Care patterns were measured at the patient level during the pre-index period for the following HCRU categories: medication use, procedure and supportive treatment use, ILD-related diagnostics use, specialist visits, provider specialty associated with index ILD diagnosis and initial ILD-related respiratory symptoms, and all-cause and respiratory-related hospitalizations and emergency department visits.

Among the subgroup of patients with ILD-related early respiratory symptoms following SARD diagnosis, the association between the timing and type of specialist (pulmonologist or rheumatologist) visit and time from the initial respiratory symptom onset to ILD diagnosis was evaluated. Patients who had a pulmonology or rheumatology visit within the first 90 days following the respiratory symptom onset were classified as having early specialist encounters. These patients were compared with those without a specialist visit within the same period. Analyses were conducted separately for each specialty, with one set of analysis for patients with and without early pulmonology visits, and another set for patients with and without early rheumatology visits.

### Statistical analysis

All study measures were analysed descriptively. Demographics were measured on index date and clinical characteristics and care patterns were measured over the length of the pre-index period. Demographics, clinical characteristics and care patterns were described with univariate statistics. Frequency and percentage were reported for categorical variables. Relevant measures of centrality and variance, such as mean, standard deviation (s.d.), median and interquartile range (IQR), were reported for continuous measures.

For the subgroup analysis evaluating the association between each specialist type and time from early respiratory symptom to ILD diagnosis, a Cox proportional hazards regression model (CPHM) was used to adjust for potential confounding factors, including demographics, comorbid conditions, and medication use. Backward elimination was used to remove pre-index comorbid conditions identified as not statistically significant in the model. Variables subject to backward elimination included respiratory and other conditions of interest and prescriptions filled during the pre-index period. A sensitivity analysis reviewing each specialist encounter during the 60 or 120 days following early respiratory symptoms was also performed (Supplementary Materials, available at *Rheumatology* online). A survival curve based on the CPHM was plotted for the time from early respiratory symptoms to ILD diagnosis between patients with and without early pulmonology visits.

## Results

The study population included 2526 patients who met all study inclusion criteria, of whom a subgroup of 1303 (51.6%) had early respiratory symptoms of ILD after initial SARD diagnosis and at least 90 days prior to the index date (ILD diagnosis). Additional details on patient attrition are provided in Supplementary Table S1, available at *Rheumatology* online.

The mean (s.d.) length of the pre-index period was 7.4 (3.3) years. Patient demographics and clinical characteristics are summarized in Table 1. The mean (s.d.) age of the overall study population on index date was 62.6 (13.6) years and 75.4% were female. The majority of patients were white non-Hispanic (NH) (68.5%), primarily covered by commercial insurance (80.0% *vs* Medicare Advantage), and predominantly located in urban regions (56.6%). The mean (s.d.) QCI score was 3.8 (2.7), and more than a quarter of the population (27.0%) had two or more SARDs of interest, with RA (62.6%) being the most common underlying SARD. Pneumonia was the most prevalent respiratory condition of interest (45.8%), followed by lower respiratory tract infections (39.5%) and chronic obstructive pulmonary disease (COPD; 37.8%) (Table 1).

**Table 1.** Demographics and clinical characteristics

	Study population (*n* = 2526)	Subgroup (*n* = 1303)
Demographics, measured on the index date		
Age, mean (s.d.), years	62.6 (13.6)	63.8 (13.3)
Female sex, *n* (%)^a^	1904 (75.4)	1011 (77.6)
Race/ethnicity, *n* (%)		
White Non-Hispanic (NH)	1730 (68.5)	923 (70.9)
Hispanic or Latino	199 (7.9)	97 (7.5)
Black or African American NH	265 (10.5)	133 (10.2)
Asian NH	82 (3.3)	27 (2.1)
Others NH	43 (1.7)	24 (1.8)
Unknown	207 (8.2)	98 (7.5)
Payer, *n* (%)		
Commercial	2021 (80.0)	1007 (77.3)
Medicare Advantage	505 (20.0)	296 (22.7)
Urbanicity of residence, *n* (%)		
Urban	1429 (56.6)	>705
Suburban	675 (26.7)	363 (27.9)
Rural	414 (16.4)	222 (17.0)
Missing	8 (0.3)	<5
Quartiles of SES index score^b^, *n* (%)		
1 (lowest)	604 (23.9)	317 (24.3)
2	590 (23.4)	304 (23.3)
3	585 (23.2)	309 (23.7)
4 (highest)	711 (28.2)	355 (27.3)
Missing	36 (1.4)	18 (1.4)
Clinical characteristics, measured during the pre-index period		
Length of pre-index observation period, years		
Mean (s.d.)	7.4 (3.3)	7.4 (3.2)
Median (IQR)	6.8 (4.9–9.1)	6.9 (5.0–9.1)
QCI score, mean (s.d.)	3.82 (2.7)	4.43 (2.8)
Number of patients with ≥2 ICD-9/10-CM codes indicating SARD diagnoses of interest during the pre-index period, *n* (%)		
Anti-neutrophilic cytoplasmic antibody vasculitis	63 (2.5)	33 (2.5)
Dermatomyositis/polymyositis	144 (5.7)	76 (5.8)
Mixed connective tissue disease	368 (14.6)	232 (17.8)
Rheumatoid arthritis	1582 (62.6)	889 (68.2)
Sjögren's syndrome	483 (19.1)	277 (21.3)
Systemic sclerosis	276 (10.9)	156 (12.0)
Systemic lupus erythematosus	468 (18.5)	277 (21.3)
Two or more different diagnosis categories	681 (27.0)	428 (32.9)
Three or more different diagnosis categories	226 (9.0)	155 (11.9)
Number of patients with at least 1 diagnosis during the pre-index period, *n* (%)		
Early respiratory symptoms of ILD		
Cough	917 (36.3)	584 (44.8)
Dyspnoea	2055 (81.4)	1282 (98.4)
Other symptoms of interest		
Chest pain	1699 (67.3)	1018 (78.1)
Fatigue	1906 (75.5)	1074 (82.4)
Loss of appetite	127 (5.0)	79 (6.1)
Respiratory-related conditions of interest		
Asthma	908 (35.9)	569 (43.7)
Chronic obstructive pulmonary disease	956 (37.8)	623 (47.8)
Coronavirus disease (COVID-19)	389 (15.4)	246 (18.9)
Cystic fibrosis	<5	<5
Hypersensitivity pneumonitis	21 (0.8)	14 (1.1)
Influenza	353 (14.0)	204 (15.7)
Lower respiratory tract infections^c^	997 (39.5)	618 (47.4)
Lung cancer	66 (2.6)	48 (3.7)
Pneumonia	1156 (45.8)	707 (54.3)
Pulmonary haemorrhage	297 (11.8)	176 (13.5)
Tuberculosis	25 (1.0)	16 (1.2)
Respiratory-related medical history		
History of tobacco use	1050 (41.6)	608 (46.7)
Other comorbid conditions of interest		
Atrial fibrillation	374 (14.8)	256 (19.7)
Coronary artery disease	911 (36.1)	582 (44.7)
Deep vein thrombosis	248 (9.8)	155 (11.9)
Dyslipidaemia	1826 (72.3)	1002 (76.9)
Gastroesophageal reflux disease	1571 (62.2)	941 (72.2)
Heart failure	600 (23.8)	439 (33.7)
Hepatitis C	32 (1.3)	18 (1.4)
Myocardial infarction	305 (12.1)	196 (15.0)
Myositis	581 (23.0)	338 (25.9)
Obesity	1443 (57.1)	819 (62.9)
Obstructive sleep apnoea	742 (29.4)	493 (37.8)
Portal vein thrombosis	10 (0.4)	8 (0.6)
Pulmonary embolism	173 (6.8)	132 (10.1)
Pulmonary hypertension	410 (16.2)	290 (22.3)
Stroke or transient ischaemic attack	353 (14.0)	227 (17.4)
Type 2 diabetes	833 (33.0)	487 (37.4)

The subgroup consists of patients with early respiratory symptoms related to ILD (i.e. cough and/or dyspnoea) following SARD diagnosis and occurring at least 90 days prior to and exclusive of the index date. Cells with values of 1–4, or cells where values 1–4 can be derived have been masked per Carelon Research privacy policies.

a <5 patients in both the study population and the subgroup had missing gender.

b The SES index is a composite SDOH measure based on seven factors (unemployment rate, poverty rate, median household income, median home value, proportion of not having high school degree, proportion with college degree, proportion of households that average one or more persons per room). The SES index score is reported in quartiles, with ‘4’ indicating a patient is in the top 25% of the SES index score and ‘1’ indicating a patient is in the bottom 25% of the SES index score.

c Includes acute bronchitis, bronchiectasis. IQR: interquartile range; QCCI: Quan–Charlson comorbidity index; SARD: systemic autoimmune rheumatic diseases; SES: Socioeconomic status.

Among the subgroup of patients with early respiratory symptoms, the mean (s.d.) age was 63.8 (13.3) years and 77.6% were female. The mean (s.d.) QCI score was 4.4 (2.8) and 32.9% of patients had two or more SARDs of interest. Most of the subgroup had evidence of dyspnoea prior to ILD diagnosis (98.4%). Other common symptoms included fatigue (82.4%) and chest pain (78.1%) (Table 1).

In the overall study cohort, the mean (s.d.) time from the initial SARD diagnosis to ILD diagnosis was 38.6 (26.9) months. Corticosteroids (81.7%) were the most frequently prescribed pharmacologic treatment (Table 2). Other SARD-ILD treatments, including conventional DMARDs (47.8%) and biologic DMARDs (25.1%), were also commonly prescribed. The majority of patients (61.8%) had at least one all-cause hospitalization prior to ILD diagnosis, while only 10.3% had respiratory-related hospitalizations. The mean (s.d.) length of stay per all-cause hospitalization was 5.6 (8.8) days.

**Table 2.** Care patterns prior to ILD diagnosis

	Study population (*n* = 2526)	Subgroup (*n* = 1303)
Time from the first observed SARD to ILD diagnosis, months		
Mean (s.d.)	38.6 (26.9)	49.6 (22.97)
Median (IQR)	39.0 (13–59)	48.0 (35–67)
SARD-ILD related pharmacological treatments^a^, number of patients with at least 1 prescription/administration, *n* (%)		
Biologic DMARD	633 (25.1)	385 (29.6)
Conventional (non-biologic) DMARD	1208 (47.8)	721 (55.3)
Corticosteroids	2065 (81.7)	1124 (86.3)
Other immunosuppressants	142 (5.6)	96 (7.4)
Other Pharmacologic treatments, number of patients with at least 1 prescription/administration, *n* (%)		
Bone resorption inhibitors	465 (18.4)	283 (21.7)
Histamine type 2-receptor antagonists (H2-blockers)	606 (24.0)	355 (27.2)
Intravenous immunoglobulin	67 (2.7)	50 (3.8)
Janus kinase inhibitors	121 (4.8)	88 (6.8)
Non-steroidal anti-inflammatory drugs	1857 (73.5)	970 (74.4)
Proton pump inhibitors	1469 (58.2)	878 (67.4)
Non-pharmacological treatments and procedures, number of patients with at least 1 claim, *n* (%)		
Lung transplant	<5	<5
Occupational therapy	1088 (43.1)	651 (50.0)
Oxygen therapy	1856 (73.5)	1047 (80.4)
Physical therapy	432 (17.1)	295 (22.6)
Plasmapheresis	13 (0.5)	6 (0.5)
Pulmonary rehabilitation	37 (1.5)	29 (2.2)
Radiation therapy—lungs	89 (3.5)	60 (4.6)
All-cause healthcare resource utilization		
≥1 hospitalization, *n* (%)	1,561 (61.8)	929 (71.3)
Annualized mean (s.d.)	0.45 (0.50)	0.52 (0.57)
LOS, days, mean (s.d.)	5.58 (8.84)	5.13 (6.58)
≥1 emergency department visit, *n* (%)	1,821 (72.1)	1,049 (80.5)
Annualized mean (s.d.)	0.61 (0.91)	0.73 (1.10)
Respiratory-related healthcare resource utilization		
≥1 respiratory-related hospitalization, *n* (%)	261 (10.3)	194 (14.9)
Annualized mean (s.d.)	0.21 (0.18)	0.22 (0.19)
LOS, days, mean (s.d.)	6.81 (12.88)	7.08 (13.29)
≥1 respiratory-related emergency department visit, *n* (%)	230 (9.1)	167 (12.8)
Annualized mean (s.d.)	0.21 (0.22)	0.22 (0.25)
ILD diagnostic tests, number of patients with at least 1 claim during the pre-index period, *n* (%)		
Chest CT	2024 (80.1)	1132 (86.9)
HRCT scan, a subset of chest CT	1491 (59.0)	869 (66.7)
Pulmonary function test	1524 (60.3)	936 (71.8)
ILD diagnostic tests, number of patients with at least 1 claim during the pre-index period extended through second ILD diagnosis, *n* (%)		
Chest CT	2283 (90.4)	1222 (93.8)
HRCT scan, a subset of chest CT	1862 (73.7)	1030 (79.1)
Pulmonary function test	1778 (79.4)	1032 (79.2)
Number of patients with at least 1 diagnostic claim in the 90 days after early respiratory symptoms (inclusive of Early Resp service date, *n* (%)		
Eligible number of patients		1303 (100)
Chest CT		341 (26.2)
HRCT scan, a subset of chest CT		163 (12.5)
Pulmonary function test		316 (24.3)

The subgroup consists of patients with early respiratory symptoms related to ILD (i.e. cough and/or dyspnoea) following SARD diagnosis and occurring at least 90 days prior to and exclusive of the index date. Cells with values of 1–4, or cells where values 1–4 can be derived have been masked per Carelon Research privacy policies.

a Biologic DMARDs: adalimumab, certolizumab, etanercept, golimumab, infliximab, tocilizumab, sarilumab, rituximab, belimumab, abatacept; conventional (non-biologic) DMARDs: chloroquine phosphate, hydroxychloroquine, leflunomide, methotrexate, sulfasalazine; corticosteroids: betamethasone, dexamethasone, hydrocortisone, methylprednisolone, prednisolone, prednisone; other immunosuppressants: azathioprine, cyclophosphamide, ciclosporin, mycophenolate mofetil, mycophenolate sodium, tacrolimus. HRCT: high-resolution CT; ILD: interstitial lung disease; LOS: length of stay.

The chest CT scan was the most used diagnostic test (80.1%). Within the study population, 59.0% of patients had undergone HRCT prior to their initial ILD diagnosis. Extending the pre-index period to encompass the second ILD diagnosis claim showed an increased proportion of patients undergoing diagnostic tests—90.4% had undergone chest CT while 73.7% had undergone HRCT (Table 2).

During the critical period of the first 90 days following the onset of early respiratory symptoms, the key diagnostic tests were infrequently performed (Table 2) for patients in the subgroup. However, a higher utilization of diagnostic tests was observed among patients who had a specialist encounter during the same period compared with those without such an encounter (Supplementary Table S2, available at *Rheumatology* online). Compared with patients without a pulmonologist encounter, patients with a pulmonologist encounter were more likely to have a chest CT (45.9% *vs* 19.9%), HRCT (26.4% *vs* 8.1%) and PFTs (55.1% *vs* 14.5%).

In the overall cohort, 24.9% and 11.1% of patients were initially diagnosed with ILD by a pulmonologist and rheumatologist, respectively (Table 3). Radiology was one of top three specialties associated with the initial ILD diagnosis. Within the subgroup cohort, primary care (19.4%), cardiology (19.3%) and radiology (17.5%) were the top three most common specialist types associated with the initial respiratory symptom (i.e. cough and/or dyspnoea) diagnoses (Table 3).

**Table 3.** Specialist encounters prior to ILD diagnosis

	Study population (*n* = 2526)	Subgroup (*n* = 1303)
Provider specialty associated with index ILD diagnosis, *n* (%)		
Pulmonology	629 (24.9)	344 (26.4)
Radiology	485 (19.2)	250 (19.2)
Facility^a^	309 (12.2)	154 (11.8)
Rheumatology	280 (11.1)	109 (8.4)
Primary care^b^	242 (9.6)	128 (9.8)
Unknown	239 (9.5)	135 (10.4)
Other^c^	171 (6.8)	82 (6.3)
Non-physician clinician	126 (5.0)	74 (5.7)
Cardiology	45 (1.8)	27 (2.1)
Provider specialty associated with the first ILD-related respiratory symptoms, *n* (%)		
Primary care^b^		253 (19.4)
Cardiology		252 (19.3)
Radiology		228 (17.5)
Pulmonology		132 (10.1)
Facility^a^		121 (9.3)
Non-physician clinician		100 (7.7)
Emergency medicine		78 (6.0)
Rheumatology		54 (4.1)
Other/unknown^d^		85 (6.6)
Rheumatologist		
≥1 rheumatologist encounter, *n* (%)	2132 (84.4)	1145 (87.9)
Annualized mean (s.d.)	2.37 (2.62)	2.88 (2.84)
Time from the first SARD diagnosis to the first following rheumatologist encounter, mean (s.d.), months	7.26 (13.89)	7.99 (14.60)
Pulmonologist		
≥1 pulmonologist encounter, *n* (%)	1825 (72.2)	1064 (81.7)
Annualized mean (s.d.)	0.91 (1.27)	1.09 (1.31)
Time from the first SARD diagnosis to the first following pulmonologist encounter, mean (s.d.), months	26.80 (25.27)	29.29 (25.41)

The subgroup consists of patients with early respiratory symptoms related to ILD (i.e. cough and/or dyspnoea) following SARD diagnosis and occurring at least 90 days prior to and exclusive of the index date.

a A provider specialty of Facility represents claims submitted by institutions such as hospitals, nursing homes, and other medical facilities such as independent testing facilities, ambulatory surgical centres, or portable X-ray suppliers.

b Internal medicine, family medicine, general practice, osteopathic manipulative medicine, paediatric medicine, geriatric medicine.

c The rest of the individual specialty providers are each <2% of the population.

d The rest of the individual specialty providers are each <1% of the population and the true unknown is eight patients. HRCT: high-resolution CT; ILD: interstitial lung disease; IQR: interquartile range; PFT: pulmonary function tests; SARD: systemic autoimmune rheumatic diseases.

**Figure 2.**
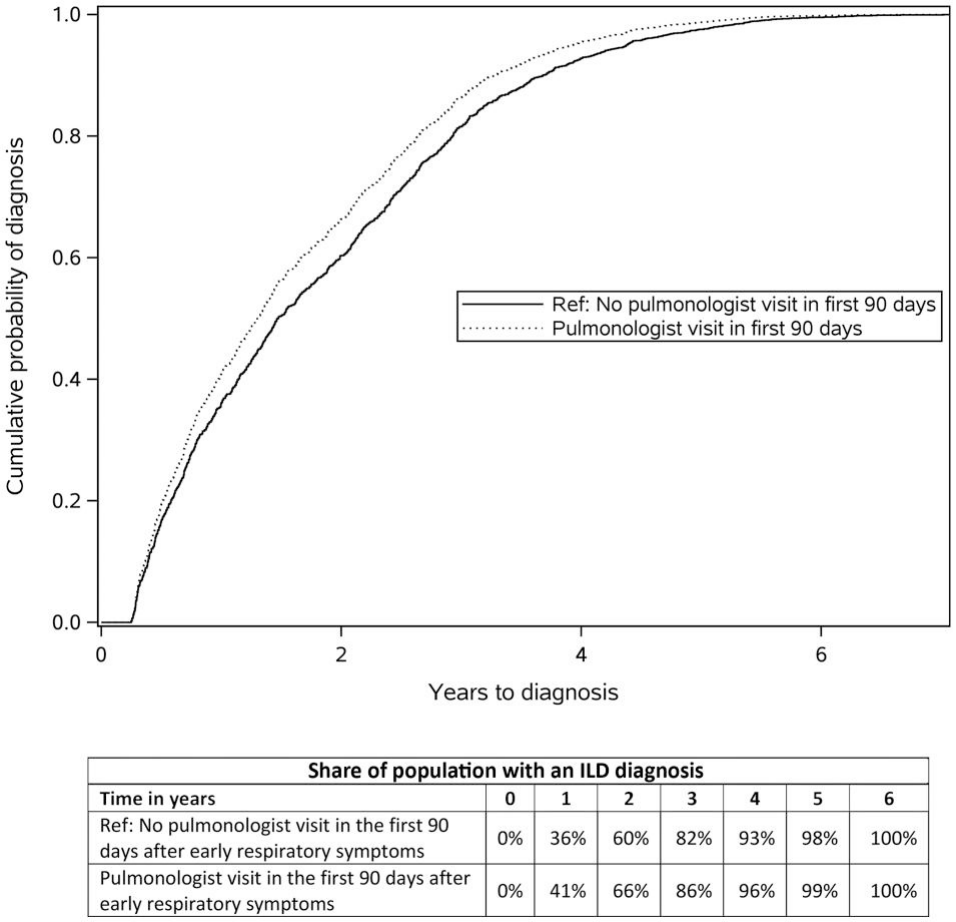
Time to ILD diagnosis for patients with or without a pulmonologist visit in the first 90 days after early respiratory symptoms. The cumulative incidence of ILD diagnosis over a 6-year period, comparing two groups based on whether they had a pulmonologist visit within the first 90 days after early respiratory symptoms. The reference group (solid line) represents individuals without pulmonologist visit within the first 90 days, while the comparison group (dotted line) represents patients with pulmonologist visit within the first 90 days. Patients who had a pulmonologist visit within 90 days were 18% more likely to be diagnosed with ILD than those without a pulmonologist encounter (hazard ratio 1.18; 95% CI: 1.03, 1.35; *P* = 0.017)

In our multivariable-adjusted CPHM, patients in the subgroup with a pulmonologist encounter in the first 90 days after initial ILD-related respiratory symptoms were, on average, 18% more likely to be diagnosed with ILD than those without a pulmonologist encounter (hazard ratio [HR]: 1.18; 95% CI: 1.03, 1.35; *P* = 0.017) (Table 4). The ILD diagnosis rate was, on average, higher for patients who had a pulmonologist encounter in the first 90 days following early respiratory symptoms onset across the time periods (Fig. 2). There was no statistically significant association between rheumatologist visit and time to ILD diagnosis (HR: 0.91; 95% CI: 0.81, 1.02; *P* = 0.114). See Supplementary Table S3, available at *Rheumatology* online for the complete Cox proportional regression model output. Results were similar in a sensitivity analysis using 60 or 120 days following respiratory diagnosis (Supplementary Table S4, available at *Rheumatology* online).

**Table 4.** Cox proportional hazards regression model for ILD diagnosis among patients with or without a specialist encounter in the first 90 days after early respiratory symptoms

Specialist encounter	HR (95%CI)	*P*-value
Pulmonologist visit within 90 days	1.18 (1.03, 1.35)	0.017
No pulmonologist visit within 90 days		Reference
Rheumatologist visit within 90 days	0.91 (0.81, 1.02)	0.114
No rheumatologist visit within 90 days		Reference

See Supplementary Table S3, available at *Rheumatology* online for the full model output and Table S4 for the sensitivity analysis. HR: hazard ratio; ILD: interstitial lung disease.

## Discussion

To our knowledge, this is the first real-world study to explore the diagnostic journey to ILD diagnosis. We described the care patterns prior to ILD diagnosis in adult patients with SARDs, including symptoms, diagnostic procedures, HCRU and specialist encounters. Furthermore, we assessed the association between an early specialist encounter following the onset of respiratory symptom and time to ILD diagnosis. Our findings underscore current unmet needs and challenges in early recognition of ILD among patients with SARDs, and potential benefits of a multidisciplinary approach to facilitate earlier SARD-ILD diagnosis.

We found that the majority of patients with SARDs who were eventually diagnosed with SARD-ILD had evidence of respiratory symptoms prior to their initial ILD diagnosis. These respiratory symptoms are non-specific and could be attributed to other comorbid conditions that were common among the study cohort, including COPD, respiratory tract infections and heart failure. This non-specificity fundamentally complicates early recognition of ILD, as these symptoms are often misattributed to decline in lung function due to older age and more prevalent chronic conditions, which often must be ruled out. Consequently, this can result in delays to diagnosis and ILD treatment initiation. Additionally, onset of respiratory symptoms is often manifested in the advanced stages of ILD [16], and thus reliance on symptom onset to initiate diagnostic evaluation would likely miss the early detection window for ILD. Therefore, these challenges are current unmet needs and underscore the importance of early and comprehensive screening of ILD in patients with SARDs to facilitate timely diagnosis.

Given the non-specificity of early symptoms, key diagnostic tools are critical to screen for and confirm the diagnosis of ILD. Our results indicate that PFTs and chest CT scans are the most frequently utilized diagnostic tools. High prevalence of these tests underscores their importance in assessing lung structure and function for accurate diagnosis. However, previous evidence suggests that they may be less adequate in detecting ILD compared with HRCT or in combination with HRCT [17, 18]. In our study, only 59.0% of the cohort underwent HRCT before or at the initial ILD diagnosis date, with utilization increasing to 73.7% when the observation period extended through the second ILD diagnosis claim. This indicates that, despite the importance of HRCT in accurately detecting ILD, it is not being used as widely as it should. This underutilization could stem from lack of standardized clinical guidelines for screening or from potential access barriers, which were not evaluated in this study. The American College of Rheumatology (ACR) and American College of Chest Physicians (CHEST) recently released guidelines for the screening and monitoring of ILD in patients with SARDs, which were not available for most of the study period covered by this investigation [5]. The guidelines conditionally recommend screening patients with SARDs at increased risk for ILD with a combination of HRCT and PFT. This screening recommendation is aligned with the European consensus statement on the identification and management of SSc-ILD and the Spanish Society of Rheumatology guidelines for the management of patients with RA [19, 20]. Therefore, the integration of these diagnostic tests into routine practice is important and the impact of the guidelines on current practices warrants future exploration.

A multidisciplinary team well-versed in the presentation and complications of SARD-ILD may facilitate early recognition of ILD. In our study, among the subset of patients with evidence of an early respiratory symptom, patients with a pulmonologist visit within the first 90 days following the symptom onset had higher utilization of diagnostic tests and a shorter time to ILD diagnosis compared with patients without a pulmonologist visit. Pulmonologists’ familiarity with, and common utilization of, key diagnostic testing for assessing and investigating lung disease could be associated with increased use of these tests leading to earlier diagnosis. This suggests that a multidisciplinary team with early involvement of a pulmonologist could expedite the time to ILD diagnosis among SARD patients with respiratory symptoms. The ACR/CHEST SARD-ILD guidelines also highlight the importance of multidisciplinary collaboration between rheumatology and pulmonology in screening for and monitoring of patients with SARD-ILD [5]. Timely and accurate diagnosis can lead to improved patient outcomes and potentially more efficient healthcare resource utilization.

There are some limitations of this study. First, there is potential for misclassification of specialist encounters billed as ‘facility’. Claims submitted by institutions such as hospitals, nursing homes and other medical facilities could have been for services provided by a rheumatologist or pulmonologist. Additionally, misclassification due to coding errors is an inherent limitation of using claims databases. Second, this study focused on patients who had a SARD diagnosis followed by an ILD diagnosis, and therefore does not consider the diagnostic pathway of those who were diagnosed with ILD prior to a SARD. Third, the claims database does not include direct clinical outcomes, such as PFT results, to assess severity of ILD, and it is possible that patients’ diagnostic journeys may differ by disease severity. However, we included and adjusted for other indicators of disease severity identifiable in claims data in our CPHM. Finally, the study results could be confounded by the COVID-19 pandemic as many outpatient visits and elective hospitalizations were delayed or cancelled, leading to a sharp decline in healthcare utilization, especially early in the pandemic [21, 22]. An important strength of this study was its use of a large cohort that is geographically varied across the U.S. including both commercially insured and Medicare Advantage members, allowing it to be generalizable to a broad population of patients with SARD-ILD.

## Conclusion

The study illustrates the complicated diagnostic journey of patients with SARD-ILD. Our findings emphasize the prevalence of non-specific symptoms prior to ILD diagnosis, which may hinder early recognition and timely intervention, potentially leading to diagnostic delays. Additionally, underutilization of essential diagnostic tests, such as HRCT scan, may contribute to diagnostic delay. Our findings suggest that involvement of a pulmonologist once a patient has ILD-related respiratory symptoms could enable earlier ILD diagnosis, and thus a multidisciplinary approach should be considered for more effective diagnosis and management of SARD-ILD. To enhance early diagnosis and improve patient outcomes, further research should focus on identifying the optimal timing for specialist involvement and fostering better coordination among specialists and other healthcare providers.

## Supplementary Material

keaf200_Supplementary_Data

## Data Availability

Due to contractual obligations to the data sources, these data are not permitted to be available for use by outside parties. The disclosure of these data to third parties assumes certain data security and privacy protocols are in place and that the third party has executed a standard license agreement which includes restrictive covenants governing the use of the data.
