# Correction to: Real-world care patterns and specialist encounters of patients with systemic autoimmune rheumatic disease-related interstitial lung disease in the United States: a retrospective administrative claims database analysis

**DOI:** 10.1093/rheumatology/keag266

**Published:** 2026-05-31

This is a correction to: Joseph Yang, Michael A Head, Kevin D Schott, Hiangkiat Tan, Rachel Djaraher, Christopher L Crowe, Mark Napier, Amy L Olson, Elana J Bernstein, Real-world care patterns and specialist encounters of patients with systemic autoimmune rheumatic disease-related interstitial lung disease in the United States: a retrospective administrative claims database analysis, *Rheumatology*, Volume 64, Issue 8, August 2025, Pages 4713–4721, https://doi.org/10.1093/rheumatology/keaf200

The following changes have been made to the originally published paper.

There has been removal of a co-author name from author list and initials from contributions list. A new **Acknowledgements** section and statement is added in the article.

