# Supplementary material for: Real-world care patterns and specialist encounters of patients with systemic autoimmune rheumatic disease-related interstitial lung disease in the United States: a retrospective administrative claims database analysis
Source: Rheumatology (Oxford). 2025 Apr 23;64(8):4713–21. doi: 10.1093/rheumatology/keaf200 (PMC12316361; doi:10.1093/rheumatology/keaf200)
Supplement: keaf200_Supplementary_Data [file keaf200_supplementary_data.docx]

**Supplementary Material**

**Supplementary Table S1: Study population identification**

| **Step** | **Criteria** | **N** | **% from previous step** |
| --- | --- | --- | --- |
| 1 | Patients with ≥ 2 SARD diagnoses of the same SARDcondition1 on different dates within 1 year, in any position and at any site of care, during the SARD identification period | 483,452 | n/a |
| 2 | Patients with ≥ 2 ILD diagnoses on different dates within 1 year, in any position and at any site of care, during the intake period | 12,379 | 3% |
| 3 | Patients with age ≥ 18 years on index date | 12,339 | 100% |
| 4 | Patients without prior ILD or antifibrotics any time pre-index (back to 2006) | 8,274 | 67% |
| 5 | Patients with ≥ 36 months of continuous medical and pharmacy insurance enrollment prior to the index date | 3,194 | 39% |
| 6 | Patient with index date (first ILD diagnosis) on or after the first observed SARD date | 2,550 | 80% |
| Study population | Patients with known geographic region | 2,526 | 99% |
| Subgroup identification | Patients with ≥ 1 diagnosis for cough and/or dyspnea after initial SARD diagnosis and >90 days prior to Index Date | 1,303 | 52% |

**Abbreviations:** N, number; SARD, Systemic autoimmune rheumatic diseases; ILD, Interstitial Lung Disease.

^1^ Antineutrophilic cytoplasmic antibody vasculitis, dermatomyositis/polymyositis, mixed connective tissue disease, rheumatoid arthritis, Sjögren’s syndrome, systemic sclerosis, systemic lupus erythematosus

**Supplementary Table S2:** Utilization of diagnostic tests in relation to specialist encounters during the first 90 days after early respiratory symptom onset

|  | **Total** | **Pulmonologist encounter during first 90 days following early respiratory symptom onset** | | **Rheumatologist encounter during first 90 days following early respiratory symptom onset** | |
| --- | --- | --- | --- | --- | --- |
|  |  | **Yes** | **No** | **Yes** | **No** |
| N (%) | 1,303 (100) | 314 (24.1) | 989 (75.9) | 595 (45.7) | 708 (54.3) |
| **Number of patients at least 1 diagnostic claim during pre-index period, n (%)** | | | | | |
| Pulmonary function test | 936 (71.8) | 275 (87.6) | 661 (66.8) | 448 (75.3) | 488 (68.9) |
| Chest CT | 1,132 (86.9) | 276 (87.9) | 856 (86.6) | 519 (87.2) | 613 (86.6) |
| HRCT scan | 869 (66.7) | 237 (75.5) | 632 (63.9) | 405 (68.1) | 464 (65.5) |
| **Number of patients at least 1 diagnostic claim in the 90-days after early respiratory symptoms (inclusive), n (%)** | | | | | |
| Pulmonary function test | 316 (24.3) | 173 (55.1) | 143 (14.5) | 148 (24.9) | 168 (23.7) |
| Chest CT | 341 (26.2) | 144 (45.9) | 197 (19.9) | 183 (30.8) | 158 (22.3) |
| HRCT scan | 163 (12.5) | 83 (26.4) | 80 (8.1) | 96 (16.1) | 67 (9.5) |

**Abbreviations:** N, number; %, percent; CT, Computed Tomography; HRCT, high-resolution computed tomography.

**Supplementary Table S3: Complete Cox proportional regression model output**

| **Characteristic** | **HR [95%CI]** | **p-value** |
| --- | --- | --- |
| **Specialist encounter** |  |  |
| Pulmonologist visit within 90 days | 1.18 [1.03 - 1.35] | 0.017 |
| No pulmonologist visit within 90 days |  | reference |
| Rheumatologist visit within 90 days | 0.91 [0.81 - 1.02] | 0.114 |
| No rheumatologist visit within 90 days |  | reference |
| **Age groups** |  |  |
| 18 to 44 | 1.94 [1.48 - 2.54] | <0.001 |
| 45 to 54 | 1.51 [1.22 - 1.87] | <0.001 |
| 55 to 64 | 1.36 [1.14 - 1.62] | 0.001 |
| 65 to 74 | 1.06 [0.89 - 1.26] | 0.535 |
| 75+ |  | reference |
| **Sex** |  |  |
| Male |  | reference |
| Female | 1.04 [0.90 - 1.19] | 0.622 |
| **Race/ethnicity** |  |  |
| White NH |  | reference |
| Hispanic or Latino | 1.27 [1.02 - 1.59] | 0.034 |
| Black or African American NH | 1.05 [0.86 - 1.29] | 0.623 |
| Asian NH | 1.46 [0.98 - 2.19] | 0.064 |
| Others NH | 1.11 [0.73 - 1.69] | 0.617 |
| Unknown | 1.11 [0.89 - 1.39] | 0.344 |
| **US geographic region** |  |  |
| Northeast |  | reference |
| Midwest | 0.95 [0.78 - 1.15] | 0.576 |
| South | 0.77 [0.64 - 0.93] | 0.006 |
| West | 0.89 [0.73 - 1.08] | 0.237 |
| **Plan type** |  |  |
| HMO |  | reference |
| PPO | 0.93 [0.81 - 1.07] | 0.313 |
| CDHP | 0.89 [0.72 - 1.11] | 0.298 |
| **Urbanicity of residence** |  |  |
| Urban |  | reference |
| Suburban | 1.06 [0.93 - 1.22] | 0.391 |
| Rural | 1.06 [0.90 - 1.24] | 0.478 |
| **Quartiles of SES index score^1^** |  |  |
| 1 (lowest) | 0.94 [0.80 - 1.11] | 0.480 |
| 2 | 0.93 [0.79 - 1.09] | 0.359 |
| 3 | 0.92 [0.79 - 1.08] | 0.331 |
| 4 (highest) |  | reference |
| **Quan-Charlson comorbidity index categories** |  |  |
| 0 | 1.51 [0.73 - 3.12] | 0.272 |
| 1 | 0.92 [0.73 - 1.15] | 0.445 |
| 2 | 1.02 [0.88 - 1.19] | 0.759 |
| 3 | 0.95 [0.79 - 1.14] | 0.589 |
| 4+ |  | reference |
| **Patients with ≥2 SARD diagnoses of interest during the pre-index period (reference is no diagnosis)** |  |  |
| Antineutrophilic cytoplasmic antibody vasculitis | 0.60 [0.41 - 0.88] | 0.008 |
| Dermatomyositis/polymyositis | 1.01 [0.79 - 1.29] | 0.945 |
| Mixed connective tissue disease | 0.86 [0.73 - 1.01] | 0.066 |
| Rheumatoid arthritis | 0.85 [0.73 - 0.99] | 0.041 |
| Sjögren's syndrome | 0.79 [0.69 - 0.92] | 0.002 |
| Systemic sclerosis | 0.79 [0.69 - 0.92] | 0.002 |
| Systemic lupus erythematosus | 0.91 [0.78 - 1.06] | 0.229 |
| **Remained in model after backward selection** |  |  |
| **Patients ≥1 comorbidity diagnosis during the pre-index period (reference is no diagnosis)** |  |  |
| Asthma | 0.76 [0.67 - 0.86] | <0.001 |
| COVID | 0.83 [0.73 - 0.95] | 0.007 |
| Cystic fibrosis | 5.45 [1.27 - 23.44] | 0.023 |
| Myositis | 0.76 [0.66 - 0.86] | <0.001 |
| Obesity | 1.28 [1.13 - 1.44] | <0.001 |
| Pulmonary hypertension | 0.86 [0.74 - 1.00] | 0.049 |
| Tuberculosis | 0.52 [0.72 - 0.98] | 0.019 |
| Stroke or transient ischemic attack | 0.84 [0.30 - 0.90] | 0.025 |
| **Patients with ≥1 prescription/administration (reference is no prescription/administration)** |  |  |
| Biologic DMARD | 0.81 [0.71 - 0.93] | 0.003 |
| Conventional (non-biologic) DMARD | 0.87 [0.77 - 0.99] | 0.030 |
| Intravenous Immunoglobulins | 0.70 [0.52 - 0.95] | 0.024 |

**Abbreviations:** NH, non-Hispanic; HMO, Health Maintenance Organization; PPO, Preferred Provider Organization; CDHP, Consumer-Driven Health Plan; SES, Socioeconomic Status; SARD, Systemic autoimmune rheumatic diseases; DMARD, Disease-modifying antirheumatic drugs.

**Supplementary Table S4: Sensitivity analysis - Cox proportional hazards regression model for ILD diagnosis among patients with or without a specialist encounter in the first 60 and 120 days after early respiratory symptoms**

| **Characteristic** | **HR [95%CI]** | **p-value** |
| --- | --- | --- |
| **Specialist encounter during first 60 days following early respiratory diagnosis** | | |
| **Specialist encounter** |  |  |
| Pulmonologist visit within 60 days | 1.28 [1.12 - 1.47] | <0.001 |
| No pulmonologist visit within 60 days |  | reference |
| Rheumatologist visit within 60 days | 0.92 [0.82 - 1.03] | 0.151 |
| No rheumatologist visit within 60 days |  | reference |
| **Specialist encounter during first 120 days following early respiratory diagnosis** | | |
| **Specialist encounter** |  |  |
| Pulmonologist visit within 120 days | 1.14 [1.00 - 1.30] | 0.057 |
| No pulmonologist visit within 120 days |  | reference |
| Rheumatologist visit within 120 days | 0.83 [0.74 - 0.93] | 0.114 |
| No rheumatologist visit within 120 days |  | reference |

**Abbreviations:** HR, hazard ratio; CI, confidence interval.
